# Letter to the Editor

**Published:** 1986-02

**Authors:** A. E. Adam

**Affiliations:** *Consultant Pathologist* Taunton and Somerset Hospital


### Letter to the Editor

Dear Sir,

My colleague Dr Jack Davies poses in your July edition the old brain teaser: 'Which is the commonest benign tumour?'

The answer surely depends on your definition of a tumour, which (like neoplasia) is far easier to describe than to define. If you will permit the simplistic version of 'a swelling' or even 'a persistent and autonomous proliferation of cells which continues after the cessation of the stimuli which caused it', then Primary FRCS candidates have nothing to fear. The answer is a blastocyst. We were all one once, even FRCS examiners.

Yours faithfully,

A. E. Adam

*Consultant Pathologist*

Taunton and Somerset Hospital

